# What’s the big idea? Identifying research priorities using linked data

**DOI:** 10.23889/ijpds.v8i2.2192

**Published:** 2023-09-14

**Authors:** Hollie Henderson, Sally Bridges, Kate Pickett, James Wilsdon

**Affiliations:** 1 University of York, York, United Kingdom; 2 Bradford Institute for Health Research, Bradford, United Kingdom; 3 University College London, London, United Kingdom

## Objectives

To develop an engagement method to identify and prioritise local evidence gaps around child and maternal health, that could be addressed using linked routinely collected data from the Born and Bred in (BaBi) studies.

## Method

We developed a pragmatic, 2-hour online workshop method to engage local stakeholders and members of the public to identify research priorities. Stakeholder groups included parents, early years practitioners, commissioners, and service providers.

The workshop involves two parts. The first part places attendees into groups with people from similar stakeholder type backgrounds to discuss areas of child and maternal health they think are important for research using linked routinely collected data. The second part places attendees into new, multidisciplinary groups to prioritise the suggestions put forward in the first part, based on criteria of urgency and importance.

## Results

A pilot workshop identified 17 important and urgent research priorities. Key themes included maternal and infant mental health, diet and childhood obesity, Covid-19, inequalities, infant feeding and labour and delivery. This engagement method has successfully been applied in five local areas that are BaBi study sites. The outputs from these workshops have informed a pipeline of projects for the BaBi Network. Research is currently being carried out that addresses one of these research priorities.

Many of the identified research priorities were not suitable for linked data research. Based on the workshop discussions, it is recommended that an understanding of the information captured in routine datasets is developed amongst stakeholders and members of the public, to allow them to better engage with linked data research.

## Conclusion

It is possible to engage a wide range of stakeholders including practitioners, commissioners, service providers and parents in a 2-hour workshop to prioritise research questions to be answered using linked routine data. This is important if research using these data is to inform local decision-making.

